# Evaluation of the relationship between circulating omentin-1 concentrations and components of the metabolic syndrome in adults without type 2 diabetes or cardiovascular disease

**DOI:** 10.1186/1758-5996-6-4

**Published:** 2014-01-15

**Authors:** Anh Vu, Maha S Sidhom, Brooke C Bredbeck, Lisa A Kosmiski, Christina L Aquilante

**Affiliations:** 1 Department of Pharmaceutical Sciences, University of Colorado Skaggs School of Pharmacy and Pharmaceutical Sciences, 12850 East Montview Boulevard, Mail Stop C238, Aurora, CO 80045 USA; 2 Division of Endocrinology, Diabetes, and Metabolism, University of Colorado School of Medicine, Aurora, CO, USA

**Keywords:** Omentin-1, Metabolic syndrome, Adipokine, Sexual dimorphism

## Abstract

**Background:**

Dysregulation of omentin-1, a beneficial adipokine, is thought to play a role in the development of type 2 diabetes and cardiovascular disease. The objective of this study was to evaluate the relationship between circulating omentin-1 concentrations and components of the metabolic syndrome in adults without type 2 diabetes or cardiovascular disease, and to determine if sex differences influenced the observed relationships.

**Methods:**

Fasting blood samples were obtained from 93 adults, ages 30–60 years, without type 2 diabetes and/or cardiovascular disease. Participants were classified as having the metabolic syndrome according to American Heart Association/National Heart, Lung and Blood Institute criteria. Plasma omentin-1 concentrations were measured using a commercially-available enzyme-linked immunosorbent assay, and relationships between plasma omentin-1 and components of the metabolic syndrome were assessed in the entire study cohort, by metabolic syndrome status, and by sex.

**Results:**

On average, participants were 48 ± 8 years of age, 50.5% were women, 54.8% were Caucasian, and 70% had the metabolic syndrome. Plasma omentin-1 concentrations did not differ significantly between individuals with versus without the metabolic syndrome (145.7 ± 70 versus 157.4 ± 79.3 ng/ml, p = 0.50). However, men with the metabolic syndrome had significantly lower omentin-1 levels than men without the metabolic syndrome (129.9 ± 66 versus 186.3 ± 84.3 ng/ml, p = 0.03). Plasma omentin-1 concentrations were significantly correlated with HDL cholesterol in the entire study cohort (r = 0.26; p = 0.01), which was primarily driven by a correlation in men (r = 0.451, p = 0.002) and participants with the metabolic syndrome (r = 0.36; p = 0.003). Plasma omentin-1 concentrations did not differ significantly between men and women; however men with the metabolic syndrome had 20% lower plasma omentin-1 levels than women with the metabolic syndrome (p = 0.06).

**Conclusion:**

These data demonstrate that circulating omentin-1 levels are associated with HDL cholesterol, primarily in men and in the presence of the metabolic syndrome. In addition, sex appears to influence the relationship between plasma omentin-1 concentrations and components of the metabolic syndrome. Additional studies are needed to explore sexual dimorphism in circulating omentin-1 levels, and the role of omentin-1 in the metabolic syndrome.

## Background

Omentin-1 is a 32 kDa adipokine that is primarily secreted by stromal vascular cells in visceral adipose tissue, and is expressed to a lesser extent in the heart, lung, and placenta [1,2]. Omentin-1 is a beneficial adipokine that enhances insulin-stimulated glucose uptake and triggers Akt signaling, which mediates downstream effects such as glucose metabolism [2,3]. Along these lines, dysregulation of omentin-1 secretion is thought to play a role in the pathophysiology of insulin resistance, inflammation, endothelial dysfunction, and cardiovascular disease [3].

In clinical studies, circulating omentin-1 concentrations have been shown to be decreased in patients with obesity, impaired glucose regulation, polycystic ovary syndrome, type 1 diabetes, and type 2 diabetes [4-10]. Low circulating levels of omentin-1 have also been associated with endothelial dysfunction and cardiovascular disease [11-16]. Given these clinical associations, omentin-1 has garnered attention as a possible contributor to the pathogenesis of the metabolic syndrome [14,17].

The metabolic syndrome is a clustering of metabolic, proinflammatory, and prothrombotic factors that increases the risk of cardiovascular disease and type 2 diabetes [18-20]. Adipose tissue dysregulation and altered secretion of numerous adipokines are present in the metabolic syndrome [20-22]. However, in regards to omentin-1, few studies have evaluated the relationship between omentin-1 and the metabolic syndrome in patients without concomitant type 2 diabetes and/or cardiovascular disease (termed “nascent metabolic syndrome” by Jialal et al.) [22,23].

The primary objective of this study was to determine the relationship between circulating omentin-1 concentrations and components of the metabolic syndrome in adults without type 2 diabetes or cardiovascular disease. We also sought to determine the influence of sex on the relationship between omentin-1 and the metabolic syndrome phenotype in these nondiabetic adults.

## Methods

### Study population

This study was conducted in nondiabetic subjects between 30 to 60 years of age who were screened for a parent metabolic syndrome clinical study at the University of Colorado. The parent study was approved by the Colorado Multiple Institutional Review Board (COMIRB 07-0817). All participants provided written informed consent for the parent study and permission to use their samples in future research.

Participants were classified as having the metabolic syndrome if they had three or more components of the American Heart Association/National Heart, Lung and Blood Institute (AHA/NHLBI) criteria: waist circumference ≥ 102 cm in men or ≥ 88 cm in women; triglycerides ≥ 150 mg/dL; high-density lipoprotein (HDL) cholesterol < 40 mg/dL in men or < 50 mg/dL in women; systolic blood pressure ≥ 130 mm Hg and/or diastolic blood pressure ≥ 85 mm Hg and/or on antihypertensive drug therapy, or fasting plasma glucose (≥ 100 mg/dL) [18].

Participants were excluded for a current or past history of cardiovascular disease, congestive heart failure, stroke or transient ischemic attack, type 1 or type 2 diabetes, gastric bypass surgery, Cushing’s syndrome, liver or kidney disease, pancreatitis, active malignancy, HIV, pregnancy, or lactation. Participants were also excluded for fasting plasma glucose ≥ 126 mg/dL, systolic blood pressure ≥ 180 mm Hg, diastolic blood pressure ≥ 110 mm Hg, triglycerides ≥ 800 mg/dL, liver enzymes ≥ 2 times the upper limit of normal, and/or serum creatinine ≥ 1.4 mg/dL. Medication exclusions included anti-diabetic medications, fibrates, nicotinic acid, systemic glucocorticoids, aspirin, non-steroidal anti-inflammatory drugs, hormonal contraceptives, estrogen hormone replacement therapy, HIV medications, calcineurin inhibitors, atypical antipsychotics, phenytoin and/or weight loss medications.

### Anthropometric and laboratory measurements

Participants were weighed in a gown and without shoes. Waist circumference was measured halfway between the lower rib and the iliac crest using a cloth tape measure. Blood pressure was measured with an automated monitor two times after a 5-minute rest period, and the average of the two blood pressures was used in study analyses. Blood samples (EDTA) were drawn in the fasting state between 7:00 AM and 11:00 AM. Blood samples were centrifuged and plasma was collected and stored at -80°C until analytical processing. Plasma omentin-1 concentrations were measured in duplicate using a commercially-available enzyme-linked immunosorbent assay (ELISA), using the protocol provided by the manufacturer (Millipore; Billerica, MA, USA). In total, five ELISA assays were run, with samples on four of the five plates being the first freeze-thaw, and samples on one plate being the second freeze-thaw. Briefly, six standard concentrations of 2 ng/ml, 4 ng/ml, 10 ng/ml, 20 ng/ml, 40 ng/ml, and 100 ng/ml were prepared. Patient samples were diluted 4-fold with assay buffer, per the protocol. Proprietary matrix solution and/or assay buffer were added to the plate. Standards, two quality controls, and patient samples were then added in duplicate to the appropriate wells. An antibody solution mixture, which contained a 1:1 mixture of capture and detection antibody, was added to each well and the plate was incubated at room temperature on an orbital shaker at moderate speed for 2 hours. After incubation, wells were decanted and washed, and an enzyme solution was added. The plate was incubated on an orbital shaker at moderate speed for 30 minutes. Following this second incubation, the wells were decanted and washed thoroughly. A substrate solution was then added to the plate and placed on an orbital shaker for 12 minutes, followed by the addition of a stop solution. Absorbance was read at 450 nm and 590 nm within five minutes. A sigmoidal 4-parameter logistic equation was used to analyze the data. The results of the patient samples were multiplied by the sample dilution factor of 4, per the manufacturer’s instructions. The lower limit of detection of the assay was 0.23 ng/ml. The intra- and inter-day coefficients of variation were 4.7% and 3.7%, respectively. Plasma glucose, total cholesterol, HDL cholesterol, and triglycerides were measured enzymatically using an Olympus AU400e Chemistry Analyzer (Olympus America Inc., Center Valley, PA, USA). LDL cholesterol was calculated from total cholesterol, HDL cholesterol, and triglycerides using the Friedewald formula [24].

### Statistical analyses

The primary endpoint of the study was the difference in plasma omentin-1 concentrations between participants with versus without the metabolic syndrome, and the correlation between plasma omentin-1 concentrations and metabolic and clinical variables in the entire study population. Data were collected and managed using Research Electronic Data Capture (REDCap) tools hosted at the University of Colorado Denver [25]. Data are presented as mean ± standard deviation or median (range). Non-normally distributed data were log-transformed prior to analysis, and then back-transformed for numerical data presentation. Pearson’s correlations were used to determine the relationship between plasma omentin-1 concentrations and components of the metabolic syndrome. Independent t tests or ANOVA were used to compare omentin-1 concentrations between 2 groups or 3 or more groups, respectively. Data were analyzed with SPSS version 20 software (IBM, New York, NY, USA). A p value <0.05 was used as the level of significance for the primary endpoint. Other comparisons (e.g., sexual dimorphism) were not corrected for multiple testing and thus should be considered exploratory in nature.

## Results

### Baseline characteristics and metabolic syndrome phenotype

The study population consisted of 93 participants without type 2 diabetes or cardiovascular disease who were screened for the parent metabolic syndrome study. Baseline demographics of the entire study cohort, and in participants with and without the metabolic syndrome, are shown in Table 1. On average, participants were 48 ± 8 years of age, 50.5% were women, and 26% were current smokers. The racial distribution of the study population was 54.8% Caucasian, 26.9% African American, 5.4% American Indian/Eskimo/Aleutian, 2.2% Asian/Pacific Islander, and 10.8% other. The ethnic make-up of the study population was 16.1% Hispanic. In the metabolic syndrome group, 11 (17%) participants were on statins and 32 (49%) participants were on antihypertensive medication(s) (n = 21 ACE/ARBs, n = 15 potassium-sparing or thiazide diuretics, n = 8 beta blockers, and n = 2 calcium channel blockers). In participants without the metabolic syndrome, none were receiving statins, and 6 (21%) were on antihypertensive medication(s) (n = 5 ACE/ARBs, n = 2 beta-blockers, n = 2 potassium-sparing or loop diuretics, and n = 0 calcium channel blockers). The median plasma omentin-1 concentration was 137.2 ng/ml and the full range was 22.8 to 376.7 ng/ml. Mean plasma omentin-1 concentrations were higher in African Americans (164.5 ± 70.2 ng/ml) than in Caucasians (138.5 ± 75.9 ng/ml), although this finding was not statistically significant (p = 0.10).

**Table 1:** Baseline demographics of study population

**Characteristic**	**Total population (n = 93)**	**Metabolic syndrome (n = 65)**	**No metabolic syndrome (n = 28)**	^***a***^ ***P value***
Omentin-1 (ng/mL)	**149.2 ± 72.7**	145.7 ± 70.0	157.4 ± 79.3	*0.50*
Sex (women/men)	**47/46**	32/33	15/13	*0.70*
Age (years)	**48 ± 8**	49 ± 8	45 ± 8	*0.02*
Current smokers	**24 (25.8%)**	16 (24.6%)	8 (12.3%)	*0.69*
BMI (kg/m^2^)	**31.3 ± 4.7**	32.1 ± 4.0	29.3 ± 5.6	*0.02*
Weight (kg)	**91.6 ± 15.6**	93.8 ± 13.4	86.4 ± 19.2	*0.01*
Systolic blood pressure (mm Hg)	**129 ± 14**	132 ± 14	121 ± 12	*0.001*
Diastolic blood pressure (mm Hg)	**83 ± 12**	85 ± 11	77 ± 13	*0.002*
Waist circumference (cm)	**106.0 ± 11.8**	108.6 ± 9.5	99.9 ± 14.4	*0.001*
Fasting plasma glucose (mg/dL)	**93 ± 9**	95 ± 10	89 ± 8	*0.001*
Impaired fasting glucose	**28 (30.1%)**	26 (40.0%)	2 (7.1%)	*0.002*
Total cholesterol (mg/dL)	**187 ± 35**	187 ± 37	185 ± 30	*0.73*
LDL cholesterol (mg/dL)	**110 ± 29**	109 ± 30	111 ± 25	*0.80*
HDL cholesterol (mg/dL)	**44 ± 11**	41 ± 10	50 ± 12	*<0.001*
Triglycerides (mg/dL)	**166 ± 83**	187 ± 80	118 ± 71	*<0.001*
White blood cell count (x 10^9^/L)	**6.5 ± 2.0**	6.4 ± 1.8	6.7 ± 2.4	*0.60*

Data are expressed as mean ± standard deviation or number (%). ^*a*^P values are for comparisons of metabolic syndrome versus no metabolic syndrome. Plasma omentin-1 concentrations, expressed as median (interquartile range), were 137.2 ng/ml (94.2 to 186.8 ng/ml) in the total population, 133.1 ng/ml (93.5 to 184.8 ng/ml) in participants with the metabolic syndrome, and 137.4 ng/ml (104.5 to 196.2 ng/ml) in participants without the metabolic syndrome.

**Figure 1:**
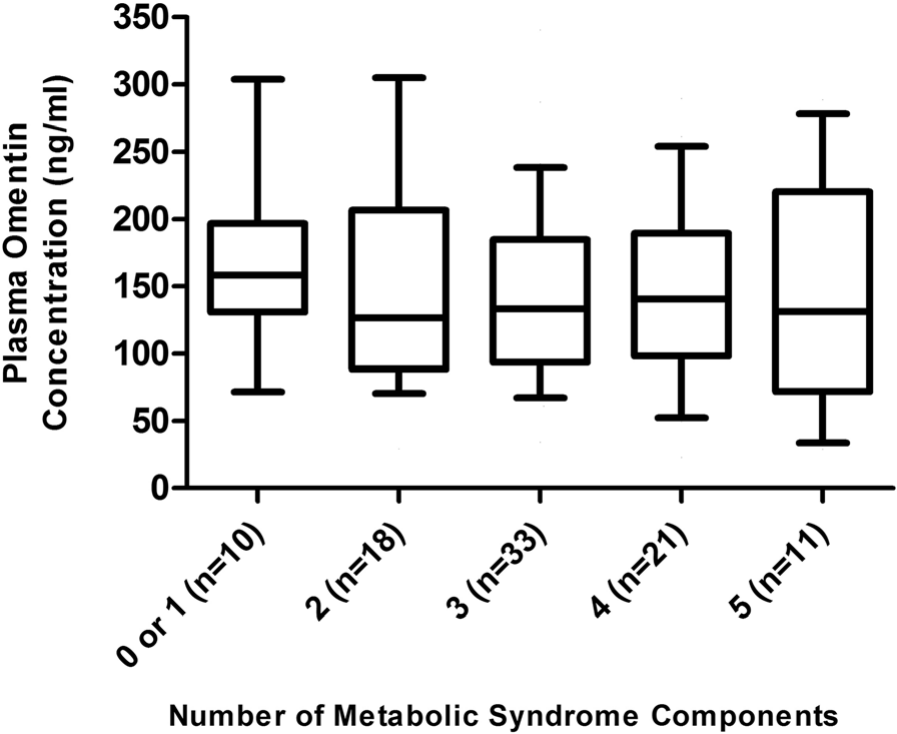
**Plasma omentin-1 concentrations according to the number of components of the metabolic syndrome.** The vertical boxes contain the interquartile range for the respective data. The horizontal solid lines in the boxes represent the median value of the data. The whiskers extending from the bottom and top of the boxes indicate the 5th and 95th percentiles of the data, respectively.

The metabolic syndrome was present in 70% of the study population, and 30% of participants had impaired fasting glucose. As expected, age, body mass index (BMI), body weight, systolic blood pressure, diastolic blood pressure, waist circumference, fasting plasma glucose, and triglycerides were significantly higher, while HDL cholesterol was significantly lower, in individuals with versus without the metabolic syndrome (Table 1). Mean plasma omentin-1 concentrations did not differ significantly between participants with versus without the metabolic syndrome (145.7 ± 70.0 ng/ml versus 157.4 ± 79.3 ng/ml, respectively; p = 0.50), nor by number of metabolic syndrome components (p = 0.82, Figure 1). Mean plasma omentin-1 concentrations were 183.1± 66.1 ng/ml in normal weight (n = 9, BMI < 25 kg/m^2^), 153.3± 88.4 ng/ml in overweight (n = 28, BMI 25.0-29.9 kg/m^2^), and 141.8± 64.1 ng/ml in obese (n = 56, BMI ≥ 30 kg/m^2^) participants (p = 0.31). In participants without the metabolic syndrome, mean plasma omentin-1 concentrations were lower in those who were overweight or obese (n = 20, 149.0 ± 83.1 ng/ml) compared with those who were normal weight (n = 8, 178.6 ± 69.1 ng/ml), although this was not statistically significant (p = 0.27). In participants with the metabolic syndrome, there was only one normal weight individual, which precluded any statistical comparisons between BMI categories. When the analysis was isolated to only overweight or obese participants, mean plasma omentin-1 concentrations did not differ significantly between those with (n = 64) versus without (n = 20) the metabolic syndrome (144.6 ± 69.9 versus 149.0 ± 83.1, respectively, p = 0.88). Mean plasma omentin-1 concentrations also did not differ significantly between non-smokers and smokers (148.5 ± 69.5 ng/ml versus 151.5 ± 82.6 ng/ml, respectively, p = 0.99). When the data were separated by smoking status, mean plasma omentin-1 concentrations did not differ significantly between non-smokers with (n = 49) versus without (n = 20) the metabolic syndrome (144.4 ± 73.1 ng/ml versus 158.3 ± 60.4 ng/ml, respectively, p = 0.23). Likewise, mean plasma omentin-1 concentrations did not differ significantly between smokers with (n = 16) versus without (n = 8) the metabolic syndrome (149.7 ± 61.3 ng/ml versus 155.1 ± 119.7 ng/ml, respectively, p = 0.56).

### Correlations between plasma omentin-1 concentrations and metabolic and clinical variables in the entire population

**Figure 2:**
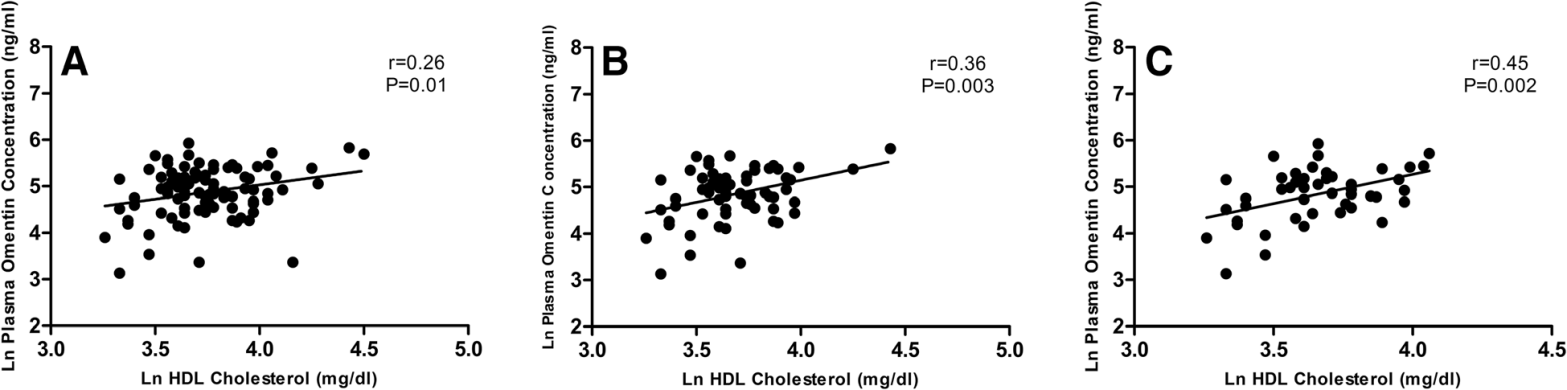
**Relationship between plasma omentin-1 concentrations and HDL cholesterol. A**. Scatterplot with regression line of the correlation between plasma omentin-1 concentrations and HDL cholesterol levels in the entire study population (n = 93). **B**. Scatterplot with regression line of the correlation between plasma omentin-1 concentrations and HDL cholesterol levels in participants with the metabolic syndrome (n = 65). **C**. Scatterplot with regression line of the correlation between plasma omentin-1 concentrations and HDL cholesterol levels in men (n = 46).

Correlations between plasma omentin-1 concentrations and components of the metabolic syndrome and clinical variables are shown in Table 2. Plasma omentin-1 concentrations were significantly correlated with HDL cholesterol in the entire study cohort (r = 0.26, p = 0.01, Figure 2A). This correlation remained significant after controlling for BMI (r = 0.25, p = 0.02). Plasma omentin-1 concentrations were also modestly correlated with triglycerides (r = -0.195) and systolic blood pressure (r = 0.198), although these results did not reach statistical significance (p = 0.06 for both variables). When the study cohort was stratified based on the presence or absence of the metabolic syndrome (Table 3), plasma omentin-1 concentrations remained significantly correlated with HDL cholesterol in participants with the metabolic syndrome (r = 0.36, p = 0.003, Figure 2B). Controlling for BMI did not change the magnitude or significance of this finding. In contrast, plasma omentin-1 concentrations were not significantly correlated with any metabolic or clinical variables in participants without the metabolic syndrome.

**Table 2:** Bivariate correlations of plasma omentin-1 concentrations and clinical/metabolic variables in the study population (n = 93)

	**Omentin-1 (ng/mL)**	
**Variable**	**Correlation coefficient (r)**	***P value***
Age (years)	0.143	*0.17*
Weight (kg)	-0.067	*0.52*
BMI (kg/m^2^)	-0.081	*0.44*
Systolic blood pressure (mm Hg)	0.198	*0.06*
Diastolic blood pressure (mm Hg)	0.109	*0.30*
Waist circumference (cm)	-0.139	*0.18*
Fasting plasma glucose (mg/dL)	0.030	*0.78*
Total cholesterol (mg/dL)	0.068	*0.52*
LDL cholesterol (mg/dL)	0.087	*0.41*
HDL cholesterol (mg/dL)	0.260	*0.01*
Triglycerides (mg/dL)	-0.195	*0.06*
White blood cell count (x 10^9^/L)	-0.063	*0.55*

**Table 3:** Bivariate correlations of plasma omentin-1 concentrations and clinical/metabolic variables by metabolic syndrome status

	**Omentin-1 (ng/mL)**		**Omentin-1 (ng/mL)**	
	**Metabolic syndrome (n = 65)**		**No metabolic syndrome (n = 28)**	
**Variable**	**Correlation coefficient (r)**	***P value***	**Correlation coefficient (r)**	***P value***
Age (years)	0.154	*0.22*	0.191	*0.33*
Weight (kg)	-0.063	*0.62*	-0.033	*0.87*
BMI (kg/m^2^)	0.015	*0.90*	-0.207	*0.29*
Systolic blood pressure (mm Hg)	0.226	*0.07*	0.270	*0.17*
Diastolic blood pressure (mm Hg)	0.144	*0.25*	0.131	*0.51*
Waist circumference (cm)	-0.110	*0.39*	-0.151	*0.44*
Fasting plasma glucose (mg/dL)	0.025	*0.84*	0.151	*0.44*
Total cholesterol (mg/dL)	0.150	*0.23*	-0.168	*0.39*
LDL cholesterol (mg/dL)	0.169	*0.18*	-0.166	*0.40*
HDL cholesterol (mg/dL)	0.360	*0.003*	-0.005	*0.98*
Triglycerides (mg/dL)	-0.179	*0.15*	-0.191	*0.33*
White blood cell count (x 10^9^/L)	-0.076	*0.55*	-0.046	*0.81*

### Relationship between plasma omentin-1 concentrations and sex

BMI and HDL cholesterol were significantly higher, while triglycerides were significantly lower, in women compared with men (Table 4). However, plasma omentin-1 concentrations did not differ significantly between men and women (Table 4). In men, plasma omentin-1 concentrations were significantly correlated with HDL cholesterol (r = 0.451, p = 0.002, Figure 2C), triglycerides (r = -0.312; p = 0.04), and BMI (r = -0.314, p = 0.03). After controlling for BMI, only HDL cholesterol remained significantly correlated with omentin-1 levels in men (r = 0.40, p = 0.006). Plasma omentin-1 concentrations were not significantly correlated with any metabolic or clinical variables in women (data not shown).

**Table 4:** Variables stratified by sex and metabolic syndrome status

	**All Women (n = 47)**	**All Men (n = 46)**	^***a***^ ***P value***	**Women with metabolic syndrome (n = 32)**	**Women without metabolic syndrome (n = 15)**	^***b***^ ***P value***	**Men with metabolic syndrome (n = 33)**	**Men without metabolic syndrome (n = 13)**	^***c***^ ***P value***
Omentin-1 (ng/mL)	152.6 ± 70.7	145.8 ± 75.2	*0.55*	162.0 ± 71.2	132.4 ± 67.6	*0.19*	129.9 ± 66.0	186.3 ± 84.4	*0.03*
Age (years)	47 ± 8	48 ± 8	*0.67*	48 ± 8	45 ± 8	*0.28*	50 ± 8	44 ± 9	*0.04*
Current smokers	11 (23.4%)	13 (28.2%)	*0.59*	7 (21.8%)	4 (26.7%)	*0.73*	9 (27.3%)	4 (30.8%)	*1.00*
BMI (kg/m^2^)	32.4 ± 4.6	30.1 ± 4.5	*0.02*	33.1 ± 4.2	31.0 ± 5.4	*0.16*	31.2 ± 3.7	27.2 ± 5.3	*0.005*
Weight (kg)	89.0 ± 15.4	94.2 ± 15.6	*0.10*	92.2 ± 15.4	82.4 ± 13.5	*0.03*	95.4 ± 11.2	91.1 ± 23.9	*0.36*
Systolic blood pressure (mm Hg)	128 ± 16	130 ± 13	*0.48*	132 ± 16	119 ± 13	*0.01*	132 ± 13	124 ± 11	*0.040*
Diastolic blood pressure (mm Hg)	81 ± 13	85 ± 11	*0.10*	83 ± 12	75 ± 14	*0.04*	87 ± 10	79 ± 11	*0.03*
Waist circumference (cm)	104.3 ± 11.1	107.7 ± 12.4	*0.16*	106.8 ± 10	98.9 ±11.6	*0.02*	110.4 ± 8.7	101.0 ± 17.4	*0.08*
Fasting plasma glucose (mg/dL)	93 ± 9	93 ± 10	*0.89*	95 ± 9	89 ± 7	*0.03*	95 ± 10	88 ± 8	*0.02*
Impaired fasting glucose	13 (27.7%)	15 (32.6%)	*0.60*	12 (37.5%)	1 (6.7%)	*0.04*	14 (42.4%)	1 (7.7%)	*0.04*
Total cholesterol (mg/dL)	184 ± 34	190 ± 36	*0.43*	185 ± 39	181 ± 22	*0.71*	190 ± 36	189 ± 39	*0.94*
LDL cholesterol (mg/dL)	107 ± 27	113 ± 30	*0.33*	108 ± 28	104 ± 23	*0.61*	110 ± 32	119 ± 26	*0.39*
HDL cholesterol (mg/dL)	47 ± 13	40 ± 8	*<0.001*	44 ± 10	55 ± 14	*0.002*	38 ± 8	44 ± 7	*0.01*
Triglycerides (mg/dL)	146 ± 79	186 ± 84	*0.02*	163 ± 80	109 ± 64	*0.01*	209 ± 75	128 ± 79	*<0.001*
White blood cell count (x 10^9^/L)	6.8 ± 2.3	6.2 ± 1.6	*0.34*	6.7 ± 2.3	7.0 ± 2.5	*0.63*	6.2 ± 1.2	6.4 ± 2.3	*0.89*

Data are expressed as mean ± standard deviation or number (%). ^a^P values for comparisons of all women versus all men. ^b^P values for comparisons of women with metabolic syndrome versus women without metabolic syndrome. ^c^P values for comparisons of men with metabolic syndrome versus men without metabolic syndrome. Plasma omentin-1 concentrations, expressed as median (interquartile range), were 133.1 ng/ml (102.2 to 212.3 ng/ml) in all women, 138.2 ng/ml (90.5 to 181.1 ng/ml) in all men, 154.0 ng/ml (109.0 to 217.9 ng/ml) in women with the metabolic syndrome, 119.7 ng/ml (89.4 to 155.4 ng/ml) in women without the metabolic syndrome, 118.9 ng/ml (77.7 to 173.1 ng/ml) in men with the metabolic syndrome, and 177.3 ng/ml (132.0 to 229.2 ng/ml) in men without the metabolic syndrome.

Men and women were further stratified based on the presence or absence of the metabolic syndrome (Table 4). Men with the metabolic syndrome had significantly lower plasma omentin-1 concentrations than men without the metabolic syndrome (p = 0.03). Men with the metabolic syndrome also had about 20% lower plasma omentin-1 levels than women with the metabolic syndrome (p = 0.06). In contrast, plasma omentin-1 concentrations did not differ significantly between women with versus without the metabolic syndrome (p = 0.19).

## Discussion

In this study, we sought to determine the relationship between circulating omentin-1 levels and components of the metabolic syndrome in adults without type 2 diabetes or cardiovascular disease. We also evaluated whether a sexual dimorphism existed for these relationships. HDL cholesterol was found to be a significant correlate of plasma omentin-1 concentrations in the entire study cohort, which was primarily driven by an association in men and in individuals with the metabolic syndrome. Circulating omentin-1 levels did not differ by metabolic syndrome status nor between men and women. When the data were stratified by sex, men with the metabolic syndrome had significantly lower omentin-1 levels than men without the metabolic syndrome and women with the metabolic syndrome. Together, these data demonstrate that omentin-1 is associated with HDL cholesterol, and that sex may influence patterns of association between omentin-1 concentrations and components of the metabolic syndrome phenotype.

The correlation between HDL cholesterol and omentin-1 has been previously described in the settings of obesity, metabolic syndrome, diabetes, and cardiovascular disease [4,8,10,12,13,17,26]. However, unlike other studies, we found that the association was primarily in men and in the presence of the metabolic syndrome. These observations are in line with previous knowledge that low HDL levels predominate in men and are a defining component of the metabolic syndrome phenotype. However, the physiologic mechanisms underlying the relationship between omentin-1 and HDL cholesterol are less clear. A plausible explanation is that dysregulation of omentin-1 may adversely affect insulin signaling and regulation, thereby altering HDL production [10,27]. A similar situation has been described for the beneficial adipokine, adiponectin [28]. Decreased adiponectin levels have been associated with decreased HDL cholesterol and an increased risk of cardiovascular disease in some studies [29-32]. Although few prospective data exist for omentin-1, some studies suggest that circulating omentin-1 concentrations are associated with atherosclerosis and coronary artery disease in different patient populations [12-14,16].

We did not observe a difference overall in circulating omentin-1 concentrations between individuals with versus without the metabolic syndrome. This finding is in contrast to Jialal et al. who reported that plasma levels of omentin-1 were 41% lower in patients with nascent metabolic syndrome as compared with controls [17]. The discrepancy is likely due to differences in study designs (e.g., inclusion and exclusion criteria) and population demographics. For example, Jialal et al. matched cases and controls on the basis of gender and age, while we did not; thus there may be more variability in our population, which may have obscured statistical significance [17]. Second, in the study by Jialal et al., 7% of patients in the metabolic syndrome group received antihypertensive medications, while in our study, 49% of patients in the metabolic syndrome group were taking antihypertensive medications. To the best of our knowledge, no clinical studies have comprehensively evaluated the effects of various antihypertensive medications on plasma omentin-1 concentrations. It is possible that the concomitant drug therapy in our study may have attenuated any associations between omentin-1 and components of the metabolic syndrome. Third, the study population by Jialal et al. was made up of 91% women, while our population was made up of 51% women. The inclusion of more men in our population may have contributed to greater variability in our population overall. Lastly, our metabolic syndrome group was younger and had a lower mean BMI than the previous study. A different study by Shibata et al. reported that circulating omentin-1 levels were inversely correlated with the number of metabolic risk factors in Japanese men who were taking no medications [33]. The demographics of that population were vastly different than ours, particularly in terms of race and concomitant medications, which may explain differences in the study findings. Taken together, compared with the published literature, our study encompasses a more heterogeneous metabolic syndrome population.

A possible sexual dimorphism in circulating omentin-1 levels has been previously reported but, to the best of our knowledge, our study is the first to compare omentin-1 levels by sex and nascent metabolic syndrome status. We observed that circulating omentin-1 did not differ between men and women in the entire study cohort; however, men with the metabolic syndrome had about 20% lower plasma omentin-1 levels than women with the metabolic syndrome. When we analyzed our data by sex, plasma omentin-1 levels were 30% lower in men with versus without the metabolic syndrome, yet did not differ between women. These findings suggest possible sex differences in omentin-1 regulation (discussed below). Other studies have reported decreased circulating omentin-1 levels in patients with the metabolic syndrome; however, direct comparisons with these studies are difficult given the presence of type 2 diabetes, cardiovascular disease, and/or use of different metabolic syndrome criteria in the populations [13,14]. de Souza Batista et al. were the first to show that, in healthy Amish subjects, women had higher circulating omentin-1 concentrations than men [4]. Moreno-Navarrete et al. also showed that sex was an independent predictor of circulating omentin-1 levels in obese subjects [34]. In contrast, other studies have found no significant differences in plasma omentin-1 levels between men and women in various clinical populations [10,13,35].

Although the relationship between sex and circulating omentin-1 concentrations is not entirely consistent between studies, it has been hypothesized that sexual dimorphism in circulating adipokines, such as omentin-1, may be a result of differences in the pattern of body fat distribution between men and women (e.g., android versus gynoid), inherent sex differences in adipose tissue gene expression and function, or the impact of sex hormones on omentin-1 regulation [10,36,37]. In this regard, Luque-Ramirez et al. recently reported that circulating omentin-1 levels were negatively correlated with free testosterone concentrations in normal weight and overweight subjects [36]. These data are consistent with situations of androgen excess (e.g., polycystic ovary syndrome), in which omentin-1 levels have been shown to be lower than controls, and inversely associated with circulating androgen levels [7,8,38,39]. Additional in vitro and in vivo studies are needed to further explore the mechanisms underlying putative sex differences in omentin-1 regulation in patients with nascent metabolic syndrome. Prospective, longitudinal studies are also needed to determine whether circulating omentin-1 concentrations are an independent and robust predictor of diabetes, subclinical inflammation, and/or cardiovascular risk [40].

### Limitations

There are limitations of our study that deserve to be acknowledged. First, our study employed a cross-sectional design; therefore, we could not evaluate the causal relationship between plasma omentin-1 levels and components of the metabolic syndrome phenotype. Second, we did not prospectively obtain insulin or adiponectin measurements in our study. Thus, we could not evaluate associations of omentin-1 with insulin resistance or adiponectin. Third, the analysis of sex differences in omentin-1 concentrations was a secondary objective of our study. As such, we did not measure sex hormones nor did we control for menopausal status. Third, we observed considerable variability in plasma omentin-1 concentrations in our study (range, 22.8 to 376.7 ng/mL). Unfortunately, few published studies have presented the full range of plasma omentin-1 concentrations in their respective populations. As such, it is difficult for us to directly compare the range of omentin-1 concentrations observed in our study with other groups. It is also important to note that some of the samples in our study were analyzed after a second freeze-thaw cycle. This may have led to omentin-1 degradation and lower omentin-1 levels in our study population. However, if we compare calculated coefficients of variation (CVs) of omentin-1 levels between studies, it appears that the omentin-1 CVs in our study were comparable to other groups. These data suggest that there is substantial variability in plasma omentin-1 concentrations. The reasons for this are unclear, but may be due to differences in laboratory methodologies and ELISA assays, patient populations, participant fat mass and distribution, concomitant medications, social factors, environmental factors, and/or genetics. Lastly, our sample size was small, which may have limited power to detect significant associations, particularly in smaller subgroups.

## Conclusions

Taken together, our data suggest that omentin-1 is associated with HDL cholesterol and that sex influences the relationship between circulating omentin-1 levels and components of the metabolic syndrome phenotype. Additional studies are needed to further explore sexual dimorphism in circulating omentin-1 levels, and the ability of omentin-1 to predict the future development of type 2 diabetes and cardiovascular disease in nondiabetic patients with the metabolic syndrome.

## Abbreviations

AHA/NHLBI: American Heart Association/National Heart, Lung, and Blood Institute; BMI: Body mass index; CV: Coefficient of variation; ELISA: Enzyme-linked immunosorbent assay; HDL: High density lipoprotein cholesterol; LDL: Low density lipoprotein cholesterol.

## Competing interests

The authors declare that they have no competing interests.

## Authors’ contributions

AV participated in the design of the study, carried out the immunoassays, participated in data collection, analysis, and interpretation, and drafted the manuscript. MS, BB, and LK participated in subject enrollment, data collection, and manuscript revision. CA conceived of the study and its design, provided oversight of subject enrollment and data collection, performed the data analysis and interpretation, and revised the manuscript. All authors read and approved the final manuscript.

## References

[B1] Schaffler A Neumeier M Herfarth H Furst A Scholmerich J Buchler C Genomic structure of human omentin, a new adipocytokine expressed in omental adipose tissue Biochim Biophys Acta 2005 1732 96 102 10.1016/j.bbaexp.2005.11.005 16386808

[B2] Yang RZ Lee MJ Hu H Pray J Wu HB Hansen BC Shuldiner AR Fried SK McLenithan JC Gong DW Identification of omentin as a novel depot-specific adipokine in human adipose tissue: possible role in modulating insulin action Am J Physiol Endocrinol Metab 2006 290 E1253 1261 10.1152/ajpendo.00572.2004 16531507

[B3] Tan BK Adya R Randeva HS Omentin: a novel link between inflammation, diabesity, and cardiovascular disease Trends Cardiovasc Med 2010 20 143 148 10.1016/j.tcm.2010.12.002 21742269

[B4] de Souza Batista CM Yang RZ Lee MJ Glynn NM Yu DZ Pray J Ndubuizu K Patil S Schwartz A Kligman M Fried SK Gong DW Shuldiner AR Pollin TI McLenithan JC Omentin plasma levels and gene expression are decreased in obesity Diabetes 2007 56 1655 1661 10.2337/db06-1506 17329619

[B5] Auguet T Quintero Y Riesco D Morancho B Terra X Crescenti A Broch M Aguilar C Olona M Porras JA Hernandez M Sabench F del Castillo D Richart C New adipokines vaspin and omentin. Circulating levels and gene expression in adipose tissue from morbidly obese women BMC Med Genet 2011 12 60 10.1186/1471-2350-12-60 21526992 PMC3107780

[B6] Pan HY Guo L Li Q Changes of serum omentin-1 levels in normal subjects and in patients with impaired glucose regulation and with newly diagnosed and untreated type 2 diabetes Diabetes Res Clin Pract 2010 88 29 33 10.1016/j.diabres.2010.01.013 20129687

[B7] Tan BK Adya R Farhatullah S Lewandowski KC O'Hare P Lehnert H Randeva HS Omentin-1, a novel adipokine, is decreased in overweight insulin-resistant women with polycystic ovary syndrome: ex vivo and in vivo regulation of omentin-1 by insulin and glucose Diabetes 2008 57 801 808 10.2337/db07-0990 18174521

[B8] Choi JH Rhee EJ Kim KH Woo HY Lee WY Sung KC Plasma omentin-1 levels are reduced in non-obese women with normal glucose tolerance and polycystic ovary syndrome Eur J Endocrinol 2011 165 789 796 10.1530/EJE-11-0375 21865408

[B9] Tan BK Pua S Syed F Lewandowski KC O'Hare JP Randeva HS Decreased plasma omentin-1 levels in Type 1 diabetes mellitus Diabet Med 2008 25 1254 1255 10.1111/j.1464-5491.2008.02568.x 19046210

[B10] Yan P Liu D Long M Ren Y Pang J Li R Changes of serum omentin levels and relationship between omentin and adiponectin concentrations in type 2 diabetes mellitus Exp Clin Endocrinol Diabetes 2011 119 257 263 10.1055/s-0030-1269912 21374544

[B11] Moreno-Navarrete JM Ortega F Castro A Sabater M Ricart W Fernandez-Real JM Circulating omentin as a novel biomarker of endothelial dysfunction Obesity (Silver Spring) 2011 19 1552 1559 10.1038/oby.2010.351 21293447

[B12] Shibata R Ouchi N Kikuchi R Takahashi R Takeshita K Kataoka Y Ohashi K Ikeda N Kihara S Murohara T Circulating omentin is associated with coronary artery disease in men Atherosclerosis 2011 219 811 814 10.1016/j.atherosclerosis.2011.08.017 21925659

[B13] Liu R Wang X Bu P Omentin-1 is associated with carotid atherosclerosis in patients with metabolic syndrome Diabetes Res Clin Pract 2011 93 21 25 10.1016/j.diabres.2011.03.001 21497934

[B14] Shang FJ Wang JP Liu XT Zheng QS Xue YS Wang B Zhao LY Serum omentin-1 levels are inversely associated with the presence and severity of coronary artery disease in patients with metabolic syndrome Biomarkers 2011 16 657 662 10.3109/1354750X.2011.622789 21988056

[B15] Yoo HJ Hwang SY Hong HC Choi HY Yang SJ Seo JA Kim SG Kim NH Choi KM Choi DS Baik SH Association of circulating omentin-1 level with arterial stiffness and carotid plaque in type 2 diabetes Cardiovasc Diabetol 2011 10 103 10.1186/1475-2840-10-103 22108456 PMC3235986

[B16] Zhong X Zhang HY Tan H Zhou Y Liu FL Chen FQ Shang DY Association of serum omentin-1 levels with coronary artery disease Acta Pharmacol Sin 2011 32 873 878 10.1038/aps.2011.26 21602837 PMC4003121

[B17] Jialal I Devaraj S Kaur H Adams-Huet B Bremer AA Increased chemerin and decreased omentin-1 in both adipose tissue and plasma in nascent metabolic syndrome J Clin Endocrinol Metab 2013 98 E514 517 10.1210/jc.2012-3673 23303213

[B18] Grundy SM Cleeman JI Daniels SR Donato KA Eckel RH Franklin BA Gordon DJ Krauss RM Savage PJ Smith SC Jr Spertus JA Costa F Diagnosis and management of the metabolic syndrome: an american heart association/national heart, lung, and blood institute scientific statement Circulation 2005 112 2735 2752 10.1161/CIRCULATIONAHA.105.169404 16157765

[B19] Alberti KG Zimmet P Shaw J The metabolic syndrome–a new worldwide definition Lancet 2005 366 1059 1062 10.1016/S0140-6736(05)67402-8 16182882

[B20] Cornier MA Dabelea D Hernandez TL Lindstrom RC Steig AJ Stob NR Van Pelt RE Wang H Eckel RH The metabolic syndrome Endocr Rev 2008 29 777 822 10.1210/er.2008-0024 18971485 PMC5393149

[B21] Bremer AA Devaraj S Afify A Jialal I Adipose tissue dysregulation in patients with metabolic syndrome J Clin Endocrinol Metab 2011 96 E1782 1788 10.1210/jc.2011-1577 21865369 PMC3205887

[B22] Bremer AA Jialal I Adipose tissue dysfunction in nascent metabolic syndrome J Obes 2013 2013 393192 23653857 10.1155/2013/393192 PMC3638696

[B23] Jialal I Devaraj S Adams-Huet B Chen X Kaur H Increased cellular and circulating biomarkers of oxidative stress in nascent metabolic syndrome J Clin Endocrinol Metab 2012 97 E1844 1850 10.1210/jc.2012-2498 22872691

[B24] Friedewald WT Levy RI Fredrickson DS Estimation of the concentration of low-density lipoprotein cholesterol in plasma, without use of the preparative ultracentrifuge Clin Chem 1972 18 499 502 4337382

[B25] Harris PA Taylor R Thielke R Payne J Gonzalez N Conde JG Research electronic data capture (REDCap)–a metadata-driven methodology and workflow process for providing translational research informatics support J Biomed Inform 2009 42 377 381 10.1016/j.jbi.2008.08.010 18929686 PMC2700030

[B26] Greulich S Chen WJ Maxhera B Rijzewijk LJ van der Meer RW Jonker JT Mueller H de Wiza DH Floerke RR Smiris K Lamb HJ de Roos A Bax JJ Romijn JA Smit JW Akhyari P Lichtenberg A Eckel J Diamant M Ouwens DM Cardioprotective properties of omentin-1 in type 2 diabetes: evidence from clinical and in vitro studies PLoS One 2013 8 e59697 10.1371/journal.pone.0059697 23555749 PMC3612072

[B27] Rashid S Watanabe T Sakaue T Lewis GF Mechanisms of HDL lowering in insulin resistant, hypertriglyceridemic states: the combined effect of HDL triglyceride enrichment and elevated hepatic lipase activity Clin Biochem 2003 36 421 429 10.1016/S0009-9120(03)00078-X 12951168

[B28] Toth PP Adiponectin and high-density lipoprotein: a metabolic association through thick and thin Eur Heart J 2005 26 1579 1581 10.1093/eurheartj/ehi374 15961408

[B29] Turer AT Scherer PE Adiponectin: mechanistic insights and clinical implications Diabetologia 2012 55 2319 2326 10.1007/s00125-012-2598-x 22688349

[B30] Pischon T Girman CJ Hotamisligil GS Rifai N Hu FB Rimm EB Plasma adiponectin levels and risk of myocardial infarction in men JAMA 2004 291 1730 1737 10.1001/jama.291.14.1730 15082700

[B31] Pischon T Hu FB Girman CJ Rifai N Manson JE Rexrode KM Rimm EB Plasma total and high molecular weight adiponectin levels and risk of coronary heart disease in women Atherosclerosis 2011 219 322 329 10.1016/j.atherosclerosis.2011.07.011 21813129 PMC3206156

[B32] Zhang H Mo X Hao Y Huang J Lu X Cao J Gu D Adiponectin levels and risk of coronary heart disease: a meta-analysis of prospective studies Am J Med Sci 2013 345 455 461 10.1097/MAJ.0b013e318262dbef 23123561

[B33] Shibata R Ouchi N Takahashi R Terakura Y Ohashi K Ikeda N Higuchi A Terasaki H Kihara S Murohara T Omentin as a novel biomarker of metabolic risk factors Diabetol Metab Syndr 2012 4 37 10.1186/1758-5996-4-37 22835063 PMC3411496

[B34] Moreno-Navarrete JM Catalan V Ortega F Gomez-Ambrosi J Ricart W Fruhbeck G Fernandez-Real JM Circulating omentin concentration increases after weight loss Nutr Metab (Lond) 2010 7 27 10.1186/1743-7075-7-27 20380714 PMC2859768

[B35] Akbarzadeh S Nabipour I Assadi M Movahed A Jafari SM Motamed N Nazem H Haraghy M Pourbehi A Bargahi A Hajian N The normoglycemic first-degree relatives of patients with type 2 diabetes mellitus have low circulating omentin-1 and adiponectin levels Cytokine 2012 58 295 299 10.1016/j.cyto.2012.02.005 22398372

[B36] Luque-Ramirez M Martinez-Garcia MA Montes-Nieto R Fernandez-Duran E Insenser M Alpanes M Escobar-Morreale HF Sexual dimorphism in adipose tissue function as evidenced by circulating adipokine concentrations in the fasting state and after an oral glucose challenge Hum Reprod 2013 28 1908 1918 10.1093/humrep/det097 23559188

[B37] Martinez-Garcia MA Montes-Nieto R Fernandez-Duran E Insenser M Luque-Ramirez M Escobar-Morreale HF Evidence for masculinization of adipokine gene expression in visceral and subcutaneous adipose tissue of obese women with polycystic ovary syndrome (PCOS) J Clin Endocrinol Metab 2013 98 E388 396 10.1210/jc.2012-3414 23337724

[B38] Mahde A Shaker M Al-Mashhadani Z Study of Omentin1 and other adipokines and hormones in PCOS patients Oman Med J 2009 24 108 118 22334855 10.5001/omj.2009.25 PMC3273948

[B39] Tan BK Adya R Farhatullah S Chen J Lehnert H Randeva HS Metformin treatment may increase omentin-1 levels in women with polycystic ovary syndrome Diabetes 2010 59 3023 3031 10.2337/db10-0124 20852028 PMC2992762

[B40] Herder C Karakas M Koenig W Biomarkers for the prediction of type 2 diabetes and cardiovascular disease Clin Pharmacol Ther 2011 90 52 66 10.1038/clpt.2011.93 21654741

